# Tyrosine Binding Protein Sites Regulate the Intracellular Trafficking and Processing of Amyloid Precursor Protein through a Novel Lysosome-Directed Pathway

**DOI:** 10.1371/journal.pone.0161445

**Published:** 2016-10-24

**Authors:** Joshua H. K. Tam, M. Rebecca Cobb, Claudia Seah, Stephen H. Pasternak

**Affiliations:** 1 J. Allyn Taylor Centre for Cell Biology, Molecular Medicine Research Group, Robarts Research Institute, Western University, London Ontario, Canada, N6A 5B7; 2 Department of Physiology and Pharmacology, Western University, London, Ontario, Canada, N6A 5B7; 3 Program in Neuroscience, Western University, London, Ontario, Canada, N6A 5B7; 4 Department of Clinical Neurological Sciences, The Schulich School of Medicine and Dentistry, Western University, London, Ontario, Canada, N6A 5B7; Torrey Pines Institute for Molecular Studies, UNITED STATES

## Abstract

The amyloid hypothesis posits that the production of β-amyloid (Aβ) aggregates leads to neurodegeneration and cognitive decline associated with AD. Aβ is produced by sequential cleavage of the amyloid precursor protein (APP) by β- and γ-secretase. While nascent APP is well known to transit to the endosomal/ lysosomal system via the cell surface, we have recently shown that APP can also traffic to lysosomes intracellularly via its interaction with AP-3. Because AP-3 interacts with cargo protein via interaction with tyrosine motifs, we mutated the three tyrosines motif in the cytoplasmic tail of APP. Here, we show that the YTSI motif interacts with AP-3, and phosphorylation of the serine in this motif disrupts the interaction and decreases APP trafficking to lysosomes. Furthermore, we show that phosphorylation at this motif can decrease the production of neurotoxic Aβ 42. This demonstrates that reducing APP trafficking to lysosomes may be a strategy to reduce Aβ 42 in Alzheimer’s disease.

## 1. Introduction

Alzheimer’s disease (AD) is characterized by the accumulation of extracellular plaques in the brains of AD patients composed of β-amyloid (Aβ) peptides. Aβ is derived from the amyloid precursor protein (APP), a type 1 transmembrane glycoprotein. To produce Aβ, APP is cleaved first by the β-secretase, which releases the soluble APPβ ectodomain, leaving a 99-residue β-carboxyl terminal fragment (βCTF). The βCTF is then cleaved by γ-secretase to produce Aβ species varying from 38–43 residues and an APP intracellular domain. Currently, the subcellular localization of these cleavage events is unclear. For example the Golgi apparatus, plasma membrane, and autophagosomes [1–4] have been implicated in Aβ production. However, many studies show that nascent APP is cleaved after endocytosis from the cell surface into endosomes and subsequently into lysosomes [2,5–8]. We have recently shown that APP can also transit directly into lysosomes from the cell surface via macropinosomes [9,10]. We have also shown that APP and γ-secretase proteins are *bona fide* resident proteins of lysosome [11–13]. Furthermore, γ-secretase has an acidic optimal pH [11], and disruption of endosomal/lysosomal pH by chloroquine or ammonium chloride decreases the production of Aβ [14–16].

Although many studies have examined the cell surface trafficking of APP, few have examined APP’s intracellular transport. The advent of photo-activatable fluorescent proteins (pa-GFP) provided a new tool to study the intracellular behavior of proteins [17–19]. Recently, we demonstrated that a paGFP tag could be used to follow the intracellular trafficking of APP from the Golgi. We discovered that APP can traffic from the Golgi to the lysosome (via an interaction with the adaptor protein, AP-3), where it is cleaved to form Aβ [20].

**Fig 1:**
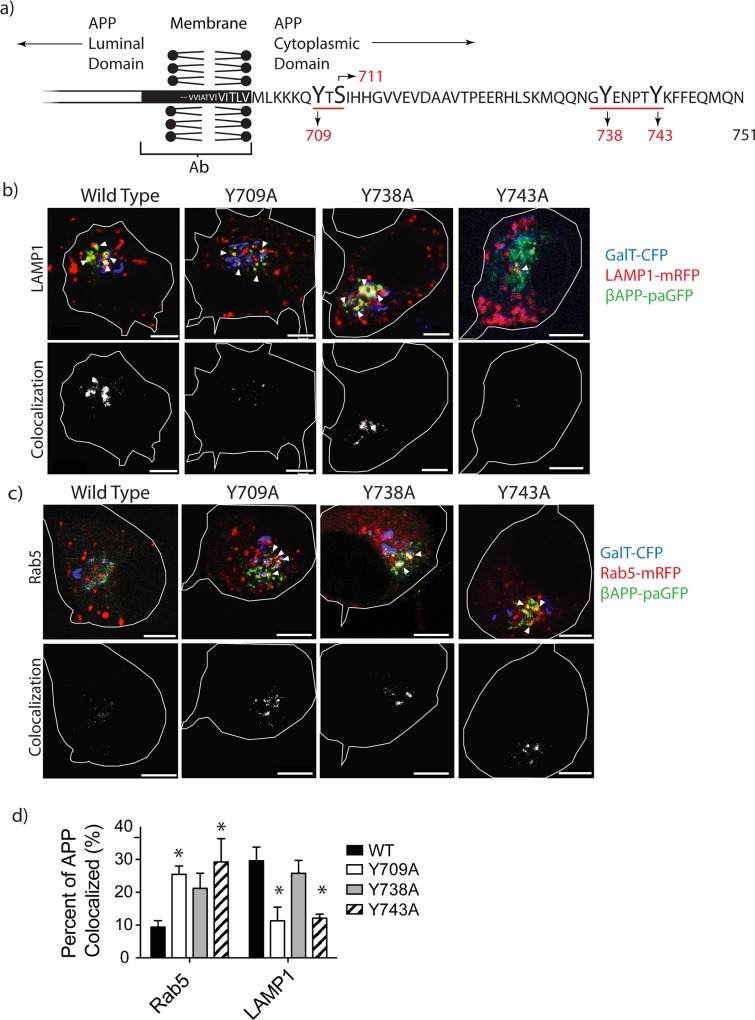
Tyrosine mutations modulate the intracellular trafficking of APP. **a)** Depiction of the carboxyl terminal of APP, with APP 751 numbering. The tyrosines and serines studied in this paper are shown, and the tyrosine motifs underlined. SN56 cells were transiently transfected with wild type APP or APP with mutations Y709A, Y738A, or Y743A tagged with paGFP. Each cell was subjected to 15-minutes of sequential imaging. Before each image, the cell was photo-activated within the Golgi (blue). **b)** Trafficking of APP to lysosomes (LAMP-1) and **c)** early endosomes (Rab5) was studied. The top panels show representative images from each cell after 15 minutes of photo-activation (Scale bars represent 5μm). The bottom panels depict colocalized pixels. The white border demarcates the edge of the cell and was drawn based on the white light images. Triangles point to colocalized pixels. **d)** Using a semi-automated method, the APP vesicles and LAMP1 or Rab5 vesicles were selected and the means were plotted using Prism 5.0b. Error bars represent SEM (* = p<0.05).

AP-3 is a heterotetrameric adaptor protein, which consists of a β3, δ3, μ3, and σ3 domains. The μ3 domain of AP-3 recognizes tyrosine motifs of the form YXXƟ (where Ɵ is a bulky amino acid and X is any amino acid) [21]. APP contains two YXXƟ motifs at ^709^YTSI^712^ and ^738^YENP^741^ (using APP 751 numbering). These motifs interact with other known members of the heterotetrameric adaptor protein family (AP-1, AP-2, and AP-4) [4,22–24]. The YENP motif is part of a larger motif that contains an NPXY motif (^738^GYENPTY^743^)(Fig 1A), which has been shown to be involved in endocytosis [6,8,25,26].

While the role of these tyrosine mutations in APP internalization is well documented, the effect of these mutations on the intracellular trafficking from the Golgi to lysosomes remains to be elucidated. Here, we use paGFP-tagged APP and live cell imaging to examine the role of these cytoplasmic tyrosine motifs on the intracellular trafficking of APP. We show that the mutation of Y709A or Y743A disrupt the transit of APP from the Golgi to the lysosome in live cells. Furthermore, the ^709^YTSI^712^ motif is responsible for the interaction of APP with AP-3. This interaction can be disrupted by phosphorylation of serine within the ^709^YTSI^712^ (S711), which can be phosphorylated by protein kinase C (PKC) [27,28], and decreases lysosomal transport from the Golgi. Furthermore, we demonstrate that PKCε activation can divert APP away from lysosomes; possibly by S711 phosphorylation.

## 2. Methods

### 2.1 Antibodies and Chemicals

Antibodies used were Mouse Anti-HA (Sigma, H9658), AP-3 (SA4, Developmental Studies Hybridoma Bank) and APP C-terminal (Sigma, A8717). The PKC activator Phorbol-12-myristate-13-acetate (PMA) was purchased from Sigma (P8139) and 8-[2-(2-pentyl-cyclopropylmethyl)-cyclopropyl]-octanoic acid (DCP-LA) was purchased from (Sigma, D5318). Staurosporine was purchased from Millipore (Cat No. 569397). Gö6976 was purchased from Tocris Bioscience (Cat. No. 2253).

### 2.2 Cell culture

SN56 cells were maintained Dulbecco’s minimal Eagle’s medium (DMEM, Invitrogen) supplemented with 10% fetal bovine serum and 50ug/ml of penicillin/streptomycin, in an incubator at 37°C with 5% CO2. Cells were split every 3–4 days; depending on confluency. SN56 cells are a cholinergic hybrid cell line, which were generated by fusing dissociated mouse septal neurons and N18TG2 neuroblastoma cells. They were chosen for our experiments because after differentiation they are cholinergic, possess neuronal morphology, and will express APP [29–31]. These were provided to us by Dr. Jane Rylett (Western University, London Ontario, Canada). In some experiments, we also examine trafficking in the N2a cell line (ATCC, Manassas, Virginia, USA). For microscopy, cells were seeded on glass-bottomed culture dishes (MatTek) one day before transfection. Cells were transfected using Lipofectamine 2000 (Invitrogen) following manufactures directions. After a 24hrs, cells were differentiated in DMEM using 1mM dibutyrl cyclic AMP (dbcAMP; Sigma) and imaged or fixed.

### 2.3 Plasmid Constructs

A plasmid encoding the last 112 amino acids of APP with paGFP and CFP C-terminal tags was previously designed [9,20]. The N-terminal of our construct is tagged with a HA epitope to facilitate cell-surface internalization experiments. Mutations were generated using a site-directed mutagenesis kit (Stratagene). Rab5-mRFP and LAMP1-mRFP were generated as previously described [9].

### 2.4 Confocal Microscopy

Images were captured with a Zeiss LSM (laser-scanning microscope) -510 META with a Zeiss 63× 1.4 numerical aperture oil immersion lens (Carl Zeiss, Oberkochen, Germany). The thickness of each optical section was set to 1 μM. Cyan fluorescent protein (CFP) was excited with a 458 nm laser and filtered with a BP 475–525 filter set. Alexa Fluor 488 and paGFP fluorescence were excited with a 488 nm laser and filtered using a band pass (BP) 500-530-nm emission filter set. Alexa Fluor 546, proximity ligation assay (PLA) red detection agent, mCherry, and mRFP fluorescence were excited with a 543 nm laser and filtered with a BP 560–615nm of LP 560nm filter set.

### 2.5 Live-cell imaging

Images were taken using a Zeiss LSM-510 META laser-scanning microscope using a Zeiss 63x 1.4 numerical aperture oil immersion lens (Carl Zeiss, Germany). Live cell imaging was performed as previously described [20,32]. Briefly, SN56 cells were washed with PBS and transferred to with pre-warmed to 37°C Hank’s Balanced Salt Solution (HBSS; Cat. No. 14025–092, Invitrogen). The confocal plates were placed on a heated stage (PeCon GmbH) connected to a Tempcontrol 37–2 digital 2-channel (PeCon GmbH), to maintain the cells at 37°C. ROIs were drawn over the Golgi apparatus as demarcated by GalT-CFP fluorescence, using the Zeiss Physiology package. As the cell can move and shift during the imaging period, the locations of these ROI were carefully monitored to ensure they remained over the Golgi for the duration of the photo-activation period. During typical experiments, the cell was alternatively imaged and photo-activated for the 15-minutes. βAPP-paGFP was photoactivated with a 25 mW 405 nm laser, set to maximum power in the pre-specified ROIs. The bleaching for each individual ROI took approximately 50msecs. There were typically 4 ROIs drawn per cell for an approximately 4sec total photoactivation time. A time delay between frames was set accordingly to photoactivate and capture an image every 30 seconds.

### 2.6 Colocalization Analysis

Colocalization analysis was performed using Imaris 7.0 Imaris Colocalization module (Biplane) as previously described [20,32]. To analyze the vesicular trafficking of βAPP in live cells, Imaris was used to create IsoSurfaces corresponding to paGFP and LAMP1-mRFP or Rab5-mRFP fluorescence, following manufacturer’s instructions. This is a semi-automated method that defines organelle distribution based on fluorescence intensity and estimated vesicle size. APP vesicles and the compartment vesicles were demarcated using Imaris, and the amount of colocalization was calculated as a percentage of material (βAPP-paGFP) within the compartment. The percentage of material value takes into account the number of pixels colocalized, as well as the intensity of each individual pixel.

For images of fixed cells, the top 2% of the brightest pixels from each channel were thresholded, and the colocalization was determined in Imaris [9]. The percentage of material colocalized was recorded and plotted in Prism Graphpad 5.0b. Prism Graphpad 5.0b was used for all graphing and statistical analysis. A One-way ANOVA was performed with a Bonferroni post-hoc test, and P values under 0.05 were considered significant.

### 2.7 Proximity Ligation Assay (PLA)

PLA was performed using a commercially available kit (Duolink; Olink Bioscience) according to manufacturer’s instructions. Briefly, cells were permeabilized with 0.01% Triton in PBS and blocked with 2% BSA/PBS and stained with primary antibodies overnight at 4°C. AP-3δ was probed with the mouse SA4 antibody (DSHB) and APP was probed with the rabbit APP C-terminal (Sigma). Cells were washed and incubated with species-specific secondary antibodies, with covalently attached single-stranded oligonucleotides. When antibodies are within 40nm, the oligonucleotides are ligated and amplified. These are then detected by fluorescent oligonucleotides.

Z-stacks were captured by confocal microscopy and the number of dots per cell was normalized to cell volume. The results were graphed using Prism and one-way ANOVA was performed with a Tukey’s post hoc test. P-values less than 0.05 were significant.

### 2.8 Internalization Assay

APP internalization was studied as preciously described [9,10]. Briefly, anti-HA antibody was labeled using a Zenon 647 labeling kit (Invitrogen), as per manufacturer’s instructions. Cells were washed with PBS and labeled with the antibody conjugate for 30 minutes on ice to tag cell-surface βAPP-CFP. The cells washed with PBS and the cells were incubated in pre-warmed HBSS at 37°C and moved to an incubator at 37°C with 5% CO_2_ for 15 minutes. After 15 minutes, cells were fixed with 4% PFA, and imaged using confocal microscopy.

### 2.9 Aβ40 and Aβ42 ELISA

SN56 cells were transfected with βAPPsw-paGFP (βAPP bearing the Swedish mutation), βAPPsw S711A-paGFP, or βAPPsw S711E-paGFP. One set of cells transfected with βAPPsw-paGFP and treated with DCP-LA. Cells were differentiated as described above and cell culture media was collected two days after differentiation. Cell culture media was centrifuged at 200 RPM for 10 minutes at 4°C to remove large cellular debris and detached cells. Aβ40 and Aβ42 were detected with the Aβ40 ELISA Kit (KHB3482) or Aβ42 Ultrasensitive ELISA Kit (KHB3544) from Life Technologies, according to manufacturer’s instructions.

## 3. Results

### 3.1 Intracellular trafficking of APP

We have previously followed the intracellular trafficking of APP-paGFP (photoactivatable GFP) from the Golgi apparatus [20]. In this paper we make use of a shortened βAPP-paGFP construct consisting of the last 112 amino acids of APP; including the β– and γ- cleavage sites fused to paGFP. In our previous experiments we demonstrated that this construct has the same trafficking pattern as full-length APP-paGFP [9,20] and undergoes β- and γ- cleavage [20]. This construct is also more easily expressed, which results in a stronger and more easily detectable fluorescence. The photo-activatable GFP (paGFP) is a form of GFP with low fluorescence after synthesis, but develops green fluorescence after irradiation with 405 nm light [18,19]. Using the βAPP-paGFP chimera we have previously demonstrated a direct trafficking pathway from the Golgi apparatus to the lysosome [20].

We had previously demonstrated that the trafficking of APP from the Golgi to lysosomes is dependent on an interaction between APP and AP-3. The interaction of AP-3 to cargo depends on cytosolic tyrosine motifs of the form YXXƟ [21]. To determine the effect of tyrosine mutations on intracellular APP trafficking, we introduced Y709A, Y738A, and Y743A mutations into βAPP-paGFP (see Fig 1A). βAPP-paGFP was transfected into SN56 cells along with the Golgi apparatus marker (Galactosyltransferase-CFP, GalT-CFP) and a marker of lysosomes (lysosome associated membrane protein 1, LAMP1-mRFP) or early endosomes (Rab5-mRFP). After differentiation, cells were transferred to a heated stage (set at 37°C) on a Zeiss LSM510 confocal microscope. Regions of interest (ROI) were then drawn on the Golgi apparatus using the GalT-CFP fluorescence as a target. Each imaging cycle consists of a brief irradiation of these the ROI with 405nm laser light at full power (25 mW for 20 iterations per imaging cycle) (for a demonstration of this technique, see videos at the Journal of Visual experimentation http://www.jove.com/video/53153/imaging-the-intracellular-trafficking-of-app-with-photoactivatable-gfp [32]), followed by imaging of the cell. These cycles were repeated over a 15-minute period (See S1 Video). In each imaging cycle, a small amount of APP-GFP was activated in the Golgi apparatus and could then be followed as it traffics to downstream compartments. The final images in these time courses are shown in Fig 1B and 1C. Using Imaris software, we set thresholds to delimit green fluorescence (photo-activated βAPP-paGFP) and red fluorescence (compartment markers) to generate a colocalization channel (Fig 1B and 1C bottom panels). We then quantitated the amount of green fluorescence co-localized with the red signal (lysosomes or early endosomes) (Fig 1D).

In these experiments, WT βAPP rapidly appeared in LAMP1-mRFP labeled compartments (29.61 ± 4.15% SEM, n = 5 independent experiments, 23 cells total) (Fig 1B and S1 Video). In our previous paper, we demonstrated that this transport was abolished by nocadazole. Therefore, this process is dependent upon microtubule-related active transport and did not occur through diffusion or accidental irradiation of endosomes or lysosomes [20,32]. It is important to note that the resolution limit of confocal microscopy does not allow us to visualize the small trafficking vesicles emanating from the Golgi. In contrast, the Y709A and Y743A mutations caused a significant decrease (p<0.05, two-way ANOVA; Bonferroni post hoc) in βAPP-paGFP co-localization with LAMP1-mRFP after 15 minutes (11.30 ± 4.180% SEM, n = 5 independent experiments, 21 cells total, and 12.12 ± 1.24% SEM, n = 3 independent experiments 10 cells total, respectively) (Fig 1B and S2 Video and S4 Video, respectively). The Y738A mutation did not significantly reduce trafficking to lysosomes as compared to WT βAPP (p>0.05, n = 4 independent experiments, 11 cells total) (Fig 1B and 1D and S3 Video).

We also analyzed cells transfected with Rab5 to determine whether tyrosine mutations shift APP into earlier compartments in the endosomal/lysosomal pathway. In these experiments, relatively small amounts of wild type βAPP trafficked to early endosomes (9.392 ± 1.956% SEM, n = 5 independent experiments, 16 cells total) (Fig 1C). In contrast, the Y709A and Y743A significantly increased trafficking to the early endosome, (25.49 ± 2.54% SEM, n = 4 independent experiments 16 cells total, and 29.260 ± 7.09% SEM, n = 3 independent experiments 11 cells total), while the Y738A mutation did not (21.23 ± 4.572% SEM, n = 4 independent experiments 16 cells total, Fig 1C and 1D) (p<0.05, two-way ANOVA; Bonferroni post hoc). Therefore, the Y709A and Y743A both reduce the intracellular trafficking of APP to lysosomes and diverts it into early endosomes.

### 3.2 APP Internalization

**Fig 2:**
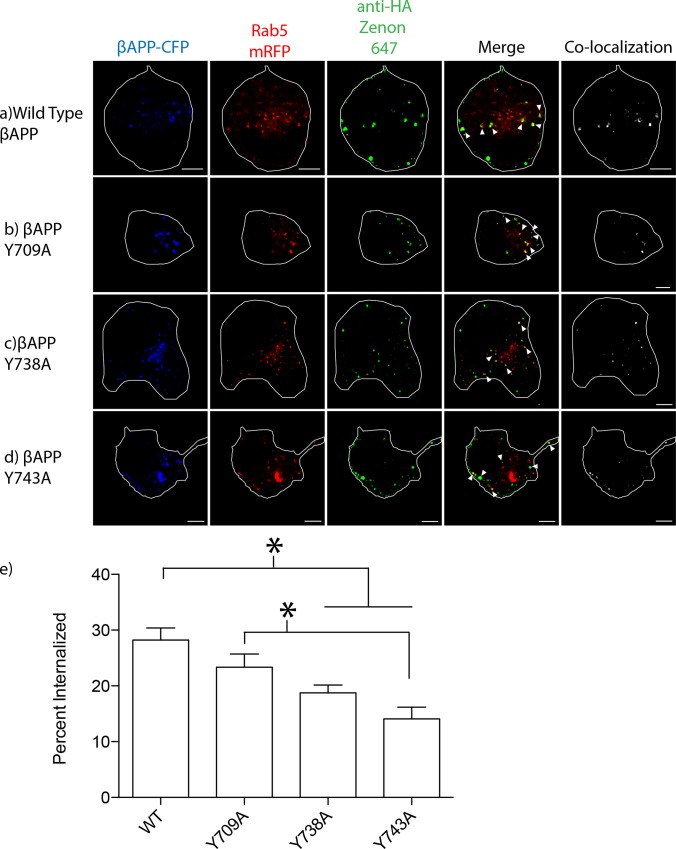
Tyrosine disrupts internalization into early endosomes. SN56 cells were transfected with βAPP-CFP (with or without tyrosine mutations) and Rab5-mRFP. The HA-tag on our βAPP-CFP construct was fluorescently labeled using an anti HA-Zenon conjugate. **a-d)** Representative images of βAPP-CFP, bearing one of the tyrosine mutations, internalized into Rab5-mRFP compartments after 15-minutes. The edge of the cell is shown by the white border, and was drawn based on white-light images. Triangles point to colocalized pixels. Scale bars represent 5μm. **e)** The percentage of APP co-localized with Rab5 was quantified using Imaris and graphed (* = p<0.05, error bars represent SEM).

Mutagenesis of tyrosine-based trafficking motifs has been shown to alter the endocytosis of APP [6,8,33]. We have shown that APP can be internalized into lysosomes by two pathways; one by way of early endosomes and a second pathway directly from the cell surface [9,10]. To determine if internalization into early endosomes was affected by mutations in C-terminal tyrosines, SN56 cells were transfected with βAPP-CFP constructs and Rab5-mRFP. Our βAPP-CFP constructs are tagged on the N-terminal with a HA epitope to facilitate internalization experiments. βAPP-CFP was surface-tagged on ice with a Zenon-647 anti-HA antibody conjugate. After a 15-minute internalization at 37°C, 28.23 ± 2.158% SEM (n = 5 independent experiments, 73 cells total) of wild type βAPP-CFP was internalized into Rab5 positive endosomes (Fig 2A). The Y709A mutation did not significantly affect the internalization of βAPP into early endosomes (23.36+/-2.338% SEM, n = 5 independent experiments, 55 cells total) (Fig 2B and 2E). However, the Y738A and Y743A mutations significantly reduced the internalization of βAPP into early endosomes (18.76+/-1.386% SEM, n = 5 independent experiments, 62 cells total and 14.09+/-2.110% SEM, n = 4 independent experiments, 53 total cells, respectively, p>0.05) (Fig 2C and 2E) [10].

**Fig 3:**
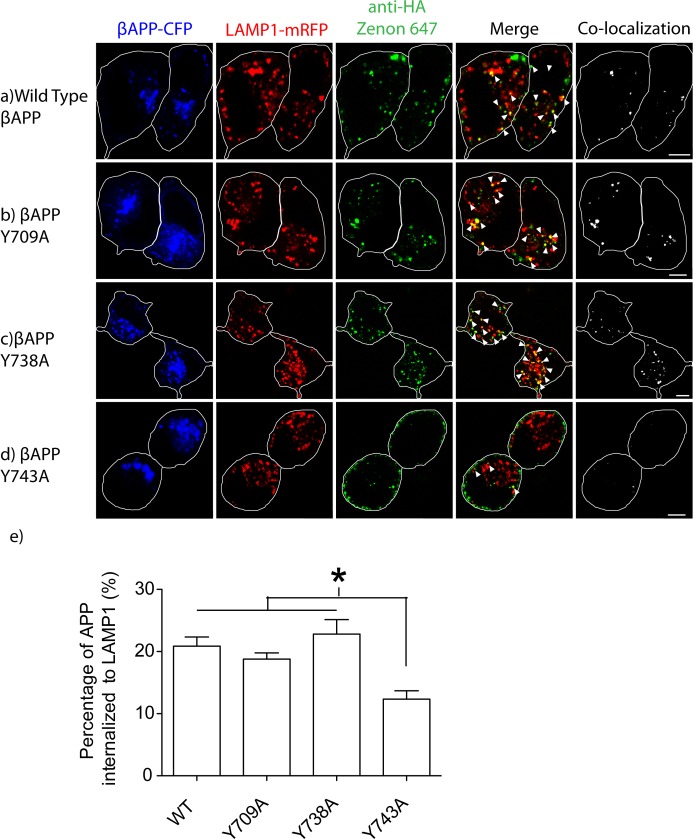
Y743A disrupts internalization into lysosomes. SN56 cells were transfected with βAPP-CFP (with or without tyrosine mutations) and Lamp1-mRFP. The HA-tag was fluorescently labeled using the anti HA-Zenon conjugate. The cells were incubated at 37°C for 15 minutes and fixed and imaged. **a-d)** Representative images of βAPP-CFP, bearing one of the tyrosine mutations, internalized into LAMP1-mRFP compartments after 15-minutes. The edge of the cell is shown by the white border, and was drawn based on white-light images. Triangles point to colocalized pixels. Scale bars represent 5μm. **e)** APP co-localized with Lamp1 was quantified using Imaris and graphed (* = p<0.05, error bars represent SEM).

To follow the direct trafficking of APP to lysosomes, SN56 cells were transfected with βAPP and LAMP1-mRFP, surface-labeled on ice with a fluorescent HA-antibody conjugate. This was followed by a 15-minute internalization period at 37°C before fixation. In cells expressing wild type βAPP, 20.87+/-1.471% SEM (n = 5 independent experiments, 83 cells total) of βAPP was internalized into LAMP1 labeled vesicles (Fig 3A and 3E). The Y709A and Y738 mutations did not significantly change the internalization of βAPP into lysosomes (p>0.05, 18.78+/-1.017% SEM, n = 5 independent experiments 70 cells total, and 22.81+/-2.328% SEM, n = 7 independent experiments 95 cells total, respectively) (Fig 3B, 3C and 3E). However, the Y743A mutation reduced internalization to 12.34 ± 1.355% SEM (p<0.05, n = 6 independent experiments 78 total cells) (Fig 3D and 3E). Therefore, the Y743A mutation disrupts βAPP internalization to both Rab5 and LAMP1 compartments, while the Y709A mutation has no effect on internalization.

### 3.3 APP/ AP-3 Interaction

Previously, we demonstrated that rapid trafficking of APP to lysosomes is dependent on APP interaction with AP-3 [20]. To determine the tyrosine motif responsible for the APP/AP-3 interaction, we performed an *in situ* proximity ligation (iPLA) assay with βAPP-CFP bearing one of the cytoplasmic tyrosine mutations. Briefly, if the proteins of interest are within 40 nm of each other, antibody-conjugated single-stranded oligonucleotides anneal and can undergo rolling circle amplification. The amplification product is detected by hybridization with fluorescent oligonucleotides, which can be visualized by confocal microscopy. iPLA has been used to confirm protein-protein interactions *in situ*, including weak or transient interactions that are undetectable by co-immunoprecipitation [34–36].

**Fig 4:**
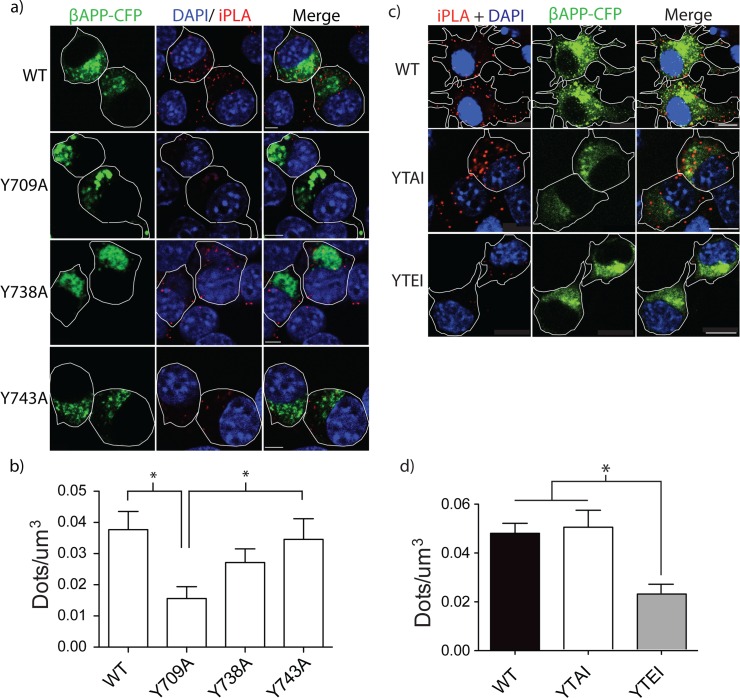
Tyrosine motif mutations affect on APP/AP-3 interaction. SN56 cells were transfected with plasmids expressing wild type APP or APP with mutations Y709A, Y738A, or Y743A. Cells were fixed and iPLA was performed to detect interaction between APP with AP-3δ. **a)** Representative images are shown. The white border shows edge of the cell and was drawn based on the white light images. Scale bars represent 5μm. **b)** The dots per cell was counted using Imaris, normalized to cell volume, and graphed in Prism 5.0b (p<0.05). SN56 cells were transfected with plasmids expressing wild type APP or APP bearing phosphomimetic (S711E) or dephosphomimetic (S711A) mutations. Cells were fixed and iPLA was performed to determine if there was an interaction between APP and AP-3. **c)** Representative images of APP/AP-3δ interaction. The white border shows edge of the cell and was drawn based on the white light images. Scale bars represent 5μm. **d)** The number of spots per cell was counted and normalized to cell volume. Error bars represent SEM and * = p<0.05.

SN56 cells were transfected with βAPP-CFP with or without tyrosine mutations and iPLA was performed after fixation. A 3D-stack of images of each cell was acquired by confocal microscopy. The βAPP-CFP /AP-3 interaction was quantified by counting the number of spots per μm3. In cells transfected with WT βAPP-CFP, cells had 0.037 ± 0.006 dots/μm^3^ SEM (n = 3 independent experiments, 28 cells total). The Y738A and Y743A mutations did not significantly alter βAPP-CFP interaction with AP-3 (0.027 ± 0.004 dots/μm3 SEM n = 3 independent experiments, 36 cells total and 0.034 ± 0.007 dots/μm3 SEM n = 3 independent experiments, 32 cells total, respectively) (Fig 4A and 4B). However, the Y709A mutation significantly decreased the interaction of βAPP-CFP with AP-3 (0.015 ± 0.004 dots/μm3 SEM, n = 3 independent experiments, 40 cells total) (Fig 4A and 4B). Therefore, it appears that the Y709A mutation disrupts the interaction of APP with AP-3 to prevent APP delivery to lysosomes.

### 3.4 Pseudo-phosphorylation of Serine 711

Having demonstrated that the Y709A mutation disrupts AP3 interaction, we sought to further characterize the function of this binding site. In the ^709^YTSI^712^ motif, the tyrosine and serine have been found to be phosphorylated in the brains of AD patients [27,37]. While the effect of Y709 phosphorylation on APP trafficking is unclear, S711 phosphorylation is pharmacologically tractable and has recently been shown to regulate the intracellular trafficking of APP [38]. Pseudo-phosphorylation of APP was shown to increase APP retrieval to the Golgi from the endosomal system and increased non-amyloidogenic processing of APP [38,39]. Furthermore, The S711 residue is the only residue in the APP C-terminus that can be phosphorylated by PKC [27,28]. To test S711 phosphorylation affects the interaction of APP with AP-3, we introduced dephosphomimetic (S711A) and phosphomimetic (S711E) mutations to the βAPP-CFP construct to determine their effect using iPLA. We show that phosphomimetic S711E-CFP interacted poorly with AP-3 (0.023 ± 0.004 dots/μm^3^ SEM, n = 4 independent experiments, 46 cells total), compared to WT βAPP-CFP (0.048 ± 0.004 dots/μm^3^ SEM, n = 4 independent experiments, 51 cells total) (Fig 4C and 4D). The dephosphomimetic (S711A) mutation did not significantly alter the interaction of βAPP with AP-3 (Fig 4C and 4D, n = 3 independent experiments, 34 cells total). Therefore, pseudo-phosphorylation of βAPP, at S711, disrupts its interaction with AP-3.

**Fig 5:**
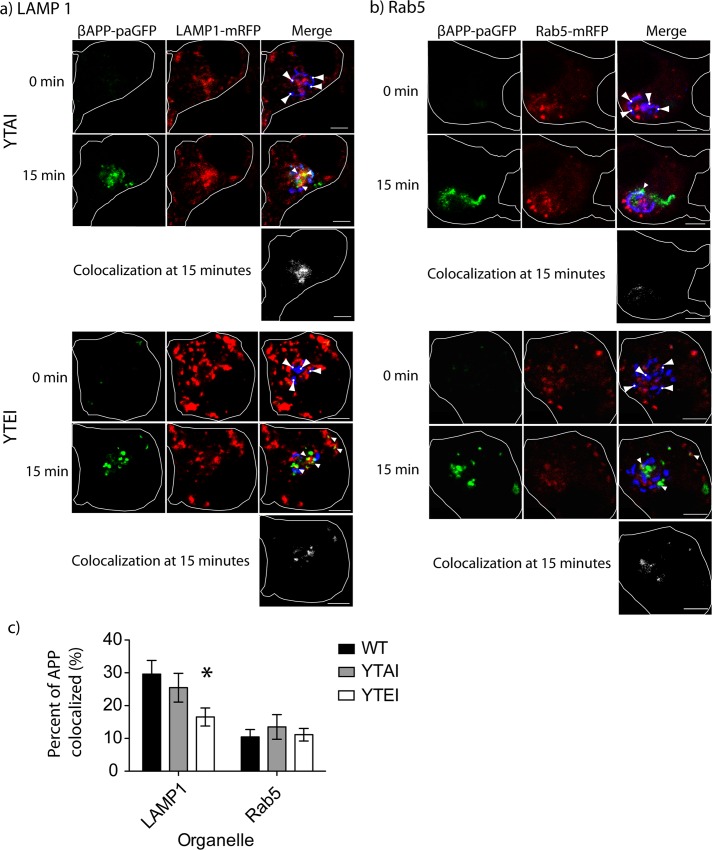
S711E disrupts trafficking to lysosomes. SN56 cells were transfected with plasmids expressing wild type APP, S711E, or S711A. Concomitantly, plasmids expressing LAMP1-mRFP or Rab5-mRFP and GalT-CFP were also transfected. **a)** Representative images depicting trafficking of APP S711A or S711E to lysosomes (LAMP1-mRFP) after photo-activation in GalT-CFP labeled compartments. **b)** Representative images showing the delivery of APP S711A or S711E to early endosomes after photo-activation. The edge of the cell is defined by the white line, and was drawn based on the white light images. Scale bars represent 5μm for all images. Triangles with circles denote photo-activation sites at time 0. Triangles alone point to colocalized pixels. **c)** The percentage of APP colocalized with either LAMP1 or Rab5 was quantified with Imaris. Error bars denote SEM. * = p<0.05.

To determine the effect of these phosphomimetic mutations on βAPP trafficking to lysosomes, we introduced the S711A and S711E mutations into our βAPP-paGFP construct. We photo-activated βAPP-paGFP in the Golgi and followed its transport into downstream compartments. The S711A mutation did not significantly disrupt βAPP trafficking to lysosomes, as compared to WT βAPP-paGFP (one-way ANOVA, n = p>0.05) (25.470±4.390% SEM n = 4 independent experiments, 13 cells total compared to 29.610±4.157% SEM, n = 5 independent experiments, 23 cells total) (Fig 5A and 5C and S5 Video). However, the S711E mutation, which disrupts βAPP interaction with AP-3, also significantly decreased the amount of βAPP trafficked to LAMP1 compartments (16.540±2.759% SEM, n = 4 independent experiments, 17 cells total), as compared to WT APP (Fig 5A and 5C and S6 Video). To determine if S711 mutations disrupts trafficking to early endosomes, we repeated the intracellular trafficking experiments with Rab5-mRFP, a marker for early endosomes. In these experiment, there was no significant difference in the amount of βAPP delivered to early endosomes with either mutation (Fig 5B and 5C). Therefore, phosphorylation of S711 impedes βAPP delivery to lysosomes likely through disrupting the βAPP/AP-3 interaction.

### 3.5 PKC activation controls intracellular trafficking of APP

S711 residue can be phosphorylated by PKC [27,28]. PKC agonists are known to increase the non-amyloidogenic processing of APP by increasing α-secretase cleavage of APP [40,41]. S711 phosphorylation has also been reported to increase the interaction of APP with members of the retromer complex, and to divert APP from the lysosome to the Golgi [38]. Phosphorylation of S711 has also been suggested to increase the secretory trafficking from the Golgi [39]. Therefore, we asked whether S711 phosphorylation could disrupt Golgi to lysosome transport, through disrupting the APP and AP-3 interaction.

**Fig 6:**
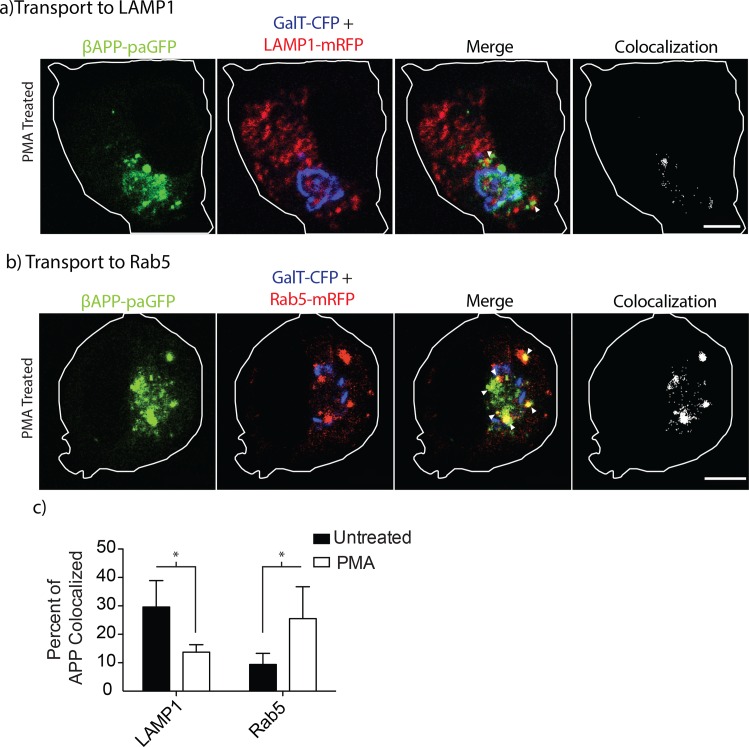
PMA treatment alters the intracellular trafficking of APP. SN56 cells transiently transfected with βAPP-paGFP were treated or not treated with 300nM PMA for 1-hour before imaging. Cells were photo-activated in the Golgi (GalT-CFP) for 15 minutes. Video of the live cells was taken during this 15-minute period to follow the trafficking of APP. Frames from the beginning and the end of the time course are shown here for transport to **a)** lysosomes (LAMP1) and **b)** early endosomes (Rab5). Far-right panels show colocalized pixels between the βAPP-paGFP and LAMP1-mRFP channels. The edge of the cell is defined by the white line, and was drawn based on the white light images. Triangles alone point to colocalized pixels. Scale bars represent 5μm for all images. **c)** The amount of APP colocalized with each compartment was quantified using Imaris at the 15-minute time point, and the results were plotted using Prism 5.0b. Error bars represent SEM and * denotes p<0.05.

To examine the effects of PKC activation on βAPP trafficking, SN56 cells were transfected with βAPP-paGFP, GalT-CFP, and a marker for the endosomes or lysosomes. After a one-hour incubation with 300nM the PKC activator Phorbol-12-myristate-13-acetate (PMA), APP was photo-activated in the Golgi apparatus to follow the transport of APP to downstream compartments (Fig 6A). In untreated cells, 29.61 ± 4.15% SEM (n = 5 independent experiments, 23 cells total) of nascent βAPP-paGFP is delivered to lysosomes. However, cells treated with 300nM PMA traffic 17.55±3.17% SEM (n = 4 independent experiments, 10 cells total) of APP to lysosomes (one-way ANOVA, Bonferroni post hoc, p<0.05) (Fig 6C). Consistent with phosphomimetic mutations to the YTSI motif, PMA treatment significantly increased the amount of βAPP-paGFP directed towards Rab5 (early endosome) labeled compartments (WT = 9.39±1.96% SEM, n = 5 independent experiments, 16 cells total vs. PMA treated 25.53±5.61% SEM, n = 5 independent experiments, 16 cells total) (Fig 6B and 6C).

**Fig 7:**
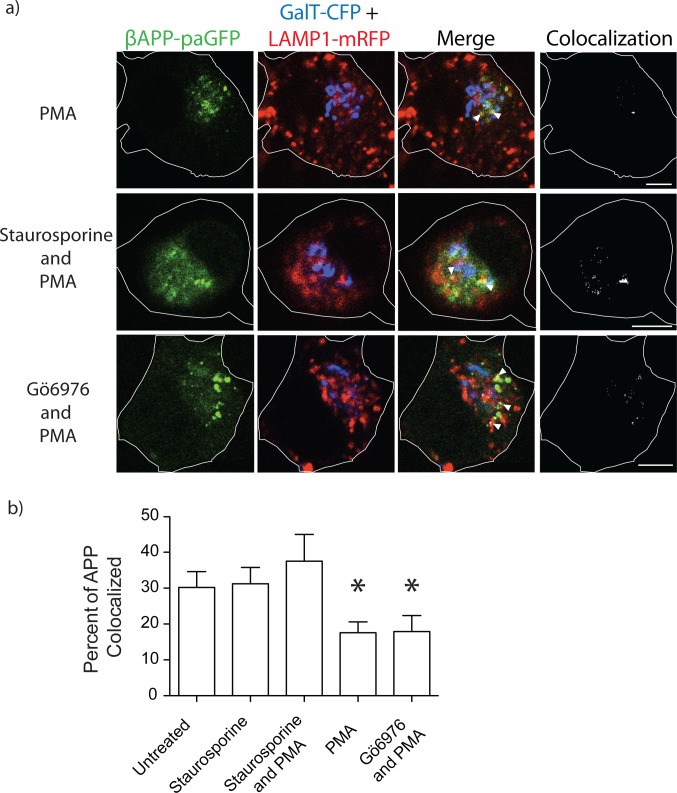
Staurosporine but not Gö6976 treatment restores trafficking of APP to lysosomes. SN56 cells were pretreated for 1 hour with staurosporine or Gö6976 for 1 hour before treatment with PMA. Cells were imaged as previously stated. Depicted in **a)** are representative images of cells treated with PMA, with or without the indicated inhibitors. These images were taken from live cell video of photo-activated cells 15 minutes after the start of imaging. Far-right panels show colocalized pixels between the βAPP-paGFP and LAMP1-mRFP channels. The edge of the cell is defined by the white line, and was drawn based on the white light images. Triangles point to colocalized pixels. Scale bars represent 5μm for all images. **b)** The amount of APP colocalized with LAMP-mRFP was quantified using Imaris and plotted using Prism. Error bars represent SEM and * denotes p<0.05 as compared to untreated cells and cells treated with staurosporine and PMA.

We also examined staurosporine treatment to inhibit PKC activity before PMA treatment. Staurosporine (1μM) pre-treatment restored the trafficking of APP to lysosomes (34.63±6.090% SEM, n = 4 independent experiments, 12 cells total) (Fig 7A and 7B). Importantly, staurosporine treatment alone did not disrupt the trafficking of APP (31.23±4.531% SEM, n = 4 independent experiments, 11 cells total). Therefore, activation of PKC diverts APP away from lysosomes and towards early endosomes.

PMA and other phorbol esters activate PKCs through binding to the diacylglycerol (DAG) binding site on PKC, and can activate conventional (α, β_I_, β_II_, and γ) and novel PKCs (δ, ε, η, and θ). PKCα and PKCε have both been suggested to regulate APP metabolism [42–45]. To specifically examine PKCα and other conventional PKC’s, we pretreated the cells with Gö6976 (inhibitor of conventional PKCs (PKCα, βI, βII, and γ). Transfected SN56 cells were pretreated with Gö6976 before stimulation with PMA. In these experiments, Gö6976 pretreatment was unable block the effects of PMA (reducing βAPP-paGFP delivery to lysosome; Gö6976 and PMA 17.97±4.056% SEM n = 3 independent experiments 10 cells total vs. PMA only 17.55±3.17% SEM n = 4 independent experiments 10 cells total) (Fig 7A and 7B). This suggests that conventional PKCs are not involved in the diverting APP away from lysosomes.

**Fig 8:**
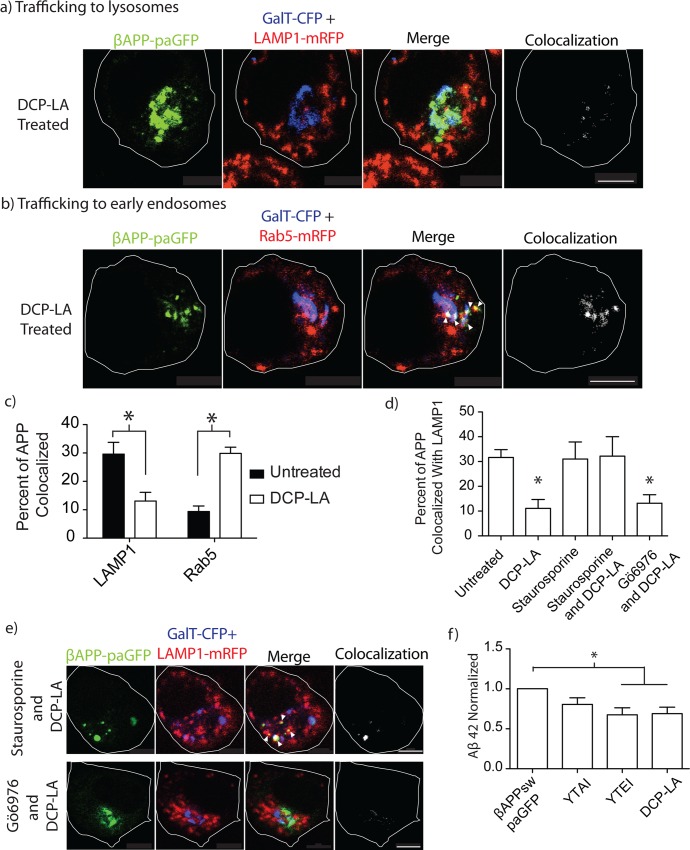
DCP-LA treatment of SN56 cells diverts APP into early endosome compartments. Cells were transfected with βAPP-paGFP, GalT-CFP, and **a)** LAMP1-mRFP or **b)** Rab5-mRFP. Cells were pre-treated with DCP-LA for one hour before imaging, and photo-activated within the Golgi. Far-right panels show colocalized pixels between the βAPP-paGFP and LAMP1-mRFP channels. The edge of the cell is defined by the white line, and was drawn based on the white light images. Triangles point to colocalized pixels. Scale bars represent 5μm. **c)** The amount of APP colocalized with each compartment, with or without DCP-LA treatement, was measured using Imaris and was plotted using Prism 5.0b. **d)** SN56 cells were treated with 1μM staurosproine or 1μM Gö6976 before treatment with DCP-LA. The amount of APP colocalized with LAMP1 was measured using Imaris and plotted using Prism 5.0b. * denotes p<0.05 as compared to untreated. Representative images from the end of the photo-activation period are shown in **e)**. Images in the far-right panel show colocalized pixels between the LAMP1-mrFP and βAPP-paGFP channels. Triangles point to colocalized pixels. Scale bars represent 5μm. **f)** SN56 cells were transfected with βAPPsw-paGFP and treated with DMSO or DCP-LA. Two other wells of cells were transfected with βAPPsw-paGFP containing either the YTEI and YTAI mutation. The media was collected from the cells and used ELISA to analyze the amount of Aβ42. Error bars in both graphs represent SEM. Results were analyzed by one-way ANOVA with a Tukey’s post hoc test. * denotes p<0.05 as compared to DMSO treated cells.

Therefore, we turned our attention to the novel PKC family. However, we could not find a specific inhibitor of the nPKCs. Instead, we turned to a specific agonist of nPKCε. Previous studies have suggested that PKCε promotes non-amyloidogenic cleavage of APP [44–46]. Recently, 8-[2-(2-pentyl-cyclopropylmethyl)-cyclopropyl]-octanoic acid (DCP-LA) was found to specifically activate PKCε over other isoforms of PKC [47]. In fact, 500nM DCP-LA has previously been shown to strongly and specifically activate PKCε and decrease Aβ production in SH-SY5Y cells [46]. We treated our transfected SN56 cells with 500nM DCP-LA, to determine if DCP-LA mediated activation of PKCε could regulate the trafficking of APP. DCP-LA significantly reduced targeting of βAPP-paGFP to lysosomes (13.09±3.04% SEM, n = 4 independent experiments, 15 cells total), which was not significantly different from cells treated with PMA (p>0.05) (Fig 8A and 8C and S7 Video). In addition, treatment of SN56 cells with DCP-LA increased delivery of APP to early endosomes, as seen with PMA treatment (Fig 8B and 8C) (WT = 9.392±1.956% SEM vs. DCP-LA treated 29.87±2.182% SEM n = 4 independent experiments, 12 cells total). Moreover, pretreatment of transfected cells with staurosporine abrogated the effect of DCP-LA on trafficking of βAPP-paGFP to lysosomes (31.94±2.111% SEM, n = 3 independent experiments, 9 cells total). However, pretreatment with Gö6976 (12.94±3.056% SEM, n = 4 independent experiments, 10 cells total) (Fig 8D and 8E) could not abolish the effects of DCP-LA treatment. These data suggest that the trafficking of APP away from lysosomes and towards early endosomes is regulated by PKCε.

To determine if PKCε can be phosphorylated at the S711 residue, we treated cells transfected with either S711E or S711A with staurosporine or DCP-LA before photoactivaton. In this indirect method, if PKCε phosphorylates APP at S711, staurosporine or DCP-LA treatment should have no effect on the trafficking of APP. When cells were transfected with S711E, there was a decrease in the amount of APP delivered to the lysosome (29.79±4.76% SEM, n = 6 independent experiments, 20 cells total). However, treatment with either staurosporine (28.75±5.24% SEM, n = 7 independent experiments, 17 cells total) or DCP-LA (30.01±8.52% SEM, n = 3 independent experiments, 8 cells total) did not significantly change the trafficking of APP as compared to the S711E mutation alone (S1 Fig). Similarly, cells transfected with S711A increased the amount of APP delivered to lysosomes (42.88±4.52% SEM, n = 6 independent experiments, 20 cells total). However, treatment with either staurosporine (36.15±7.86% SEM, n = 3 independent experiments, 9 cells total) or DCP-LA (42.96±4.89% SEM, n = 10 independent experiments, 20 cells total) did not significantly change APP trafficking in comparison with S711A alone (S1 Fig). Therefore, the main effect of PKCε activation is through phosphorylation at the S711 motif.

PKCε activation by DCP-LA can decrease Aβ production and reduce amyloid deposition in mice [44,46]. To determine if we could recapitulate these results, we transfected cells with βAPP-paGFP bearing the Swedish familial mutation (βAPPsw-paGFP) and treated the cells with DCP-LA. Cells were also transfected with either βAPPsw-paGFP YTAI or βAPPsw-paGFP YTEI to determine if psuedophosphorylation at S711 could modulate Aβ production. Cell-culture media was gathered from three independent experiments and analyzed by ELISA for Aβ 40 or 42. Aβ 42 production was not significantly reduced by transfection with APP bearing the YTAI mutation. However, transfection of APP bearing the YTEI mutation or treatment with DCP-LA significantly decreased Aβ 42 by ~30% (Fig 8F). Therefore, it appears that phosphorylation of APP at S711 decreases the production of Aβ 42 by reducing lysosomal trafficking of APP. There was no significant change in Aβ40 secreted into culture media.

## 4. Discussion

In our previous study, we showed that siRNA-mediated knockdown of AP-3 can disrupt the trafficking of APP to lysosomes [20]. In the present study, the Y709A decreased intracellular trafficking to the lysosome while the Y743A mutations significantly decreased the fraction of APP delivered to lysosomes from the cell surface and by intracellular trafficking (Figs 1–3). The YTSI motif was critical for APP and AP-3 interaction (Fig 4), and phosphorylating the serine residue in this motif reduced intracellular trafficking of APP to lysosomes and reduced Aβ42 production (Figs 5–8).

The C-terminal of APP has been shown to bind to a number of adaptor proteins, which act to regulate the function of APP. From the adaptor protein family, APP has been shown to bind to AP-1, AP-2, AP-3, and AP-4 [20,22,23,48]. AP-1 was shown to be important in the basolateral sorting of APP in Madin-Darby Canine Kidney cells [23]. In one study, AP-4 has been shown to be important in regulating the trafficking of APP at the Golgi [22]. The best studied adaptor is AP-2, which is critical in clathrin-mediated endocytosis (CME). Immunoprecipitation studies have shown that AP-2 can bind to APP [4,24,48]. Modifying the motifs that interact with AP-2 in the APP C-terminal have been shown to decrease internalization and decrease the amount of Aβ produced [8]. Recently, we have shown that APP can interact with AP-3, which directs APP to lysosomes for processing into Aβ [20]. In these experiments, we extend our findings to implicate the YTSI motif in the APP-AP-3 interaction.

The YTSI motif is a canonical YXXθ motif. These motifs have roles in endocytosis, lysosomal sorting, basolateral sorting, and retrograde sorting to the Golgi [21]. The YTSI motif has been shown to regulate the endocytosis of a APP- transferrin receptor chimera [6]. However, internalization experiments with APP show that the Y709A mutation did not disrupt endocytosis ([8] and S1 Fig). The YTSI motif can also interact with AP-1 to sort APP to the basolateral membrane [23].

Interestingly, recent studies have shown that the YTSI motif of APP can also regulate the transit of APP through the Golgi [38,39]. Using a pseudophosphorylation strategy, similar to the one used here, a phosphomimetic (S711E) increased the retrograde trafficking of APP to the TGN, and decreases the trafficking of APP lysosomes. Conversely, a dephosphomimetic mutant decreased retrograde trafficking to the TGN and increases trafficking of APP to lysosomes. The enhanced retrograde trafficking of APP to the TGN was mediated by an enhanced interaction between APP and VPS-35 (a member of the retromer protein trafficking complex) [49,50]; reducing APP delivery to lysosomes. Our findings concur with this data, in that pseudophosphorylation of the serine disrupts the interaction of APP and AP-3 and lowers the amount of APP trafficked to lysosomes (Figs 4 and 5). These findings suggest that phosphorylation of APP at S711 enhances the interaction of APP with the retromer complex [38] and destabilize its interaction with AP-3.

Phorbol ester stimulation of PKC is well known to increase the secretion of the APP N-terminal domain and decrease the production of Aβ [42,43,45,46,51]. Both PKCα and PKCε have been implicated in regulating the metabolism of APP [43,46,52,53]. In agreement with these findings, PMA or DCP-LA treatment reduced lysosomal trafficking seen with the phosphomimetic S711E (Figs 6 and 8). Gö6976, an inhibitor of conventional PKCs, did not reduce lysosomal targeting (Fig 7). While there was no specific pharmacological inhibitor of PKCε, a specific agonist of PKCε (DCP-LA) also diverted APP trafficking away from lysosomes (Fig 8). Furthermore, DCP-LA treatment or the phosphomimetic YTEI lowered the production of Aβ42, suggesting a shift to non-amyloidogenic processing of APP (Fig 8F). Previous literature suggests PKCε, an novel PKC, promotes non-amyloidogenic cleavage of APP [44–46]. DCP-LA also decreased Aβ secreted in cell culture [46], and reduce the plaque burden in transgenic mouse models of APP [44]. Furthermore, in AD patients, PKCε protein levels were decreased fibroblasts and neurons [54].

While we show here that phosphorylation of S711 may control intracellular lysosomal trafficking, it does not explain all of the observed behaviors related to PKC activation. Specifically, the phosphomimetic did not increase APP trafficking to early endosomes, as seen with PMA and DCP-LA treatments, which suggests other targets of PKCε are also involved in APP sorting. PKC is known to regulate other steps in protein trafficking and proteolysis. PKC can also phosphorylate AP-2 in the μ2 domain and regulate the endocytosis of NA+/K+ ATPase [55].

PKCε, in particular, may also regulate secretory activity from the Golgi after being recruited to the Golgi apparatus [56]. In addition to regulation of Golgi export to the secretory pathway, PKCε also regulates the recycling of β1-integrins by phosphorylating vimentin (an integral part of intermediate filaments) [57,58]. While the data presented here suggest a role for APP phosphorylation in lysosomal trafficking and non-amyloidogenic metabolism, PKCs can interact with a large number of proteins; so many other regulatory events might be participating. For example, PKCs may influence the distribution of ADAM-10; a putative α-secretase [59] and are proposed to regulate proteolytic processing by secretase enzymes directly. ADAM 10 and 17 [60,61].

Although the alteration of APP processing by PKC has long been recognized, the effects of PKC on the intracellular trafficking of APP are less well understood. Before the advent of photo-activatable fluorescent proteins, the intracellular trafficking of APP, of any protein, was very difficult to visualize. Here, using paGFP that APP can transit directly from the Golgi directly to to lysosomes. Furthermore, we show that PKCε redirects APP from this novel pathway away from the lysosome and reduces Aβ 42 production. This is the mirror image of retromer dysfunction in AD, which is proposed to increased APP levels in the endosomal/lysosomal pathway and increased Aβ production [62]. These experiments demonstrate that this novel direct-to lysosome pathway can be regulated pharmacologically and that reducing APP transit to the lysosome is a strategy to lower Aβ production.

## Supporting Information

S1 Fig PKCε activation does not control the trafficking of APP with S711E or S711A mutations. Cells were transfected with plasmids expressing S711E or S711A and a marker for lysosomes (LAMP1-mRFP) Before photoactivation, cells were pretreated with DCP-LA or staurosporine, as described earlier. APP was photo-activated in the Golgi with 405nm light, alternating with imaging for 15 minutes. The percentage of APP colocalized with either LAMP1 was quantified with Imaris. The percentage of APP colocalized was plotted in Graphpad Prism. Error bars represent SEM.
(PDF)

S1 Video APP is trafficked rapidly to lysosomes from the Golgi. SN56 cells were transiently transfected with βAPP-paGFP (green), LAMP1-mRFP (lysosome marker, red), and GalT-CFP (Golgi marker, blue). APP was photo-activated in the Golgi (blue) with 405nm light, alternating with imaging for 15 minutes (indicated by green word ‘Photo-activating’). The white circles appearing over the Golgi denote the initial ROIs for βAPP-paGFP photoactivation. These ROIs are carefully monitored and adjusted to remain on the Golgi during the photoactivation period. Cells were then chased imaging every 30 seconds for the indicated time.
(MOV)

S2 Video APP bearing the Y709A mutation does not traffic to lysosomes. SN56 cells were transiently transfected with βAPP Y709A-paGFP (green), LAMP1-mRFP (lysosome marker, red), and GalT-CFP (Golgi marker, blue). APP was photo-activated in the Golgi (blue) with 405nm light, alternating with imaging for 15 minutes (indicated by green word ‘Photo-activating’). White circles appearing over the Golgi denote the initial ROIs for βAPP-paGFP photoactivation. Cells were then chased by imaging every 30 seconds for the indicated time.
(MOV)

S3 Video APP bearing the Y738A mutation traffics to lysosomes. SN56 cells were transiently transfected with βAPP Y738A-paGFP (green), LAMP1-mRFP (lysosome marker, red), and GalT-CFP (Golgi marker, blue). APP was photo-activated in the Golgi (blue) with 405nm light, alternating with imaging for 15 minutes (indicated by green word ‘Photo-activating’). Photo-activation ROIs in the Golgi are denoted by white circles in the video. Cells were then chased by imaging every 30 seconds for the indicated time.
(MOV)

S4 Video APP bearing the Y743A mutation does not traffic to lysosomes. SN56 cells were transiently transfected with βAPP Y743A-paGFP (green), LAMP1-mRFP (lysosome marker, red), and GalT-CFP (Golgi marker, blue). APP was photo-activated in the Golgi (blue) with 405nm light, alternating with imaging for 15 minutes (indicated by green word ‘Photo-activating’). White circles appearing over the Golgi denote the initial ROIs for βAPP-paGFP photoactivation. Cells were then chased by imaging every 30 seconds for the indicated time.
(MOV)

S5 Video APP bearing the dephosphomimetic (S711A) mutation traffics to lysosomes. SN56 cells were transiently transfected with βAPP S711A-paGFP (green), LAMP1-mRFP (lysosome marker, red), and GalT-CFP (Golgi marker, blue). APP was photo-activated in the Golgi (blue) with 405nm light, alternating with imaging for 15 minutes (indicated by green word ‘Photo-activating’). The white circles appearing over the Golgi denote the initial ROIs for βAPP-paGFP photoactivation. Cells were then chased by imaging every 30 seconds for the indicated time. This movie has been intentionally cropped to focus on the trafficking around the Golgi.
(MOV)

S6 Video APP bearing the phosphomimetic (S711E) mutation does not traffic to lysosomes. SN56 cells were transiently transfected with βAPP S711E-paGFP (green), LAMP1-mRFP (lysosome marker, red), and GalT-CFP (Golgi marker, blue). APP was photo-activated in the Golgi (blue) with 405nm light, alternating with imaging for 15 minutes (indicated by green word ‘Photo-activating’). Photo-activation ROIs in the Golgi are denoted by white circles in the video. Cells were then chased by imaging every 30 seconds for the indicated time. This movie has been intentionally cropped to focus on the trafficking around the Golgi.
(MOV)

S7 Video DCP-LA treatment disrupts APP trafficking to the lysosome. SN56 cells were transiently transfected with βAPP-paGFP (green), LAMP1-mRFP (lysosome marker, red), and GalT-CFP (Golgi marker, blue) and treated with 500nM DCP-LA. APP was photo-activated in the Golgi (blue) with 405nm light, alternating with imaging for 15 minutes (indicated by green word ‘Photo-activating’). White circles in the video denote photo-activation ROIs in the Golgi. Cells were then chased imaging every 30 seconds for the indicated time.
(MOV)

## Abbreviations

APP: Amyloid precursor protein
PKC: protein kinase C
LAMP1: lysosomal membrane protein 1
paGFP: photo-activatable GFP

## References

[1] Greenfield JP, Tsai J, Gouras GK, Hai B, Thinakaran G, Checler F, et al Endoplasmic reticulum and trans-Golgi network generate distinct populations of Alzheimer beta-amyloid peptides. Proc Natl Acad Sci USA. 1999;96: 742–747. 9892704 10.1073/pnas.96.2.742 PMC15207

[2] Cirrito JR, Kang J-E, Lee J, Stewart FR, Verges DK, Silverio LM, et al Endocytosis Is Required for Synaptic Activity-Dependent Release of Amyloid-β In Vivo. Neuron. 2008;58: 42–51. 10.1016/j.neuron.2008.02.003 18400162 PMC2390913

[3] Petanceska SS, Seeger M, Checler F, Gandy S. Mutant presenilin 1 increases the levels of Alzheimer amyloid beta-peptide Abeta42 in late compartments of the constitutive secretory pathway. Journal of Neurochemistry. 2000;74: 1878–1884. 10800930 10.1046/j.1471-4159.2000.0741878.x

[4] Tian Y, Chang JC, Fan EY, Flajolet M, Greengard P. Adaptor complex AP2/PICALM, through interaction with LC3, targets Alzheimer's APP-CTF for terminal degradation via autophagy. Proceedings of the National Academy of Sciences. 2013 10.1073/pnas.1315110110 24067654 PMC3801056

[5] Thinakaran G, Koo EH. Amyloid Precursor Protein Trafficking, Processing, and Function. Journal of Biological Chemistry. 2008;283: 29615–29619. 10.1074/jbc.R800019200 18650430 PMC2573065

[6] Lai A, Sisodia SS, Trowbridge IS. Characterization of sorting signals in the beta-amyloid precursor protein cytoplasmic domain. J Biol Chem. 1995;270: 3565–3573. 7876092

[7] Lai A, Sisodia SS, Trowbridge IS. Characterization of sorting signals in the beta-amyloid precursor protein cytoplasmic domain. J Biol Chem. 1995;270: 3565–3573. 7876092

[8] Perez RG, Soriano S, Hayes JD, Ostaszewski B, Xia W, Selkoe DJ, et al Mutagenesis identifies new signals for beta-amyloid precursor protein endocytosis, turnover, and the generation of secreted fragments, including Abeta42. J Biol Chem. 1999;274: 18851–18856. 10383380 10.1074/jbc.274.27.18851

[9] Lorenzen A, Samosh J, Vandewark K, Anborgh PH, Seah C, Magalhaes AC, et al Rapid and Direct Transport of Cell Surface APP to the Lysosome defines a novel selective pathway. Mol Brain. 2010;3: 11 10.1186/1756-6606-3-11 20409323 PMC2868040

[10] Tang W, Tam JH, Seah C, Chiu J, Tyrer A, Cregan SP, et al Arf6 controls beta-amyloid production by regulating macropinocytosis of the Amyloid Precursor Protein to lysosomes. Mol Brain. 2015;8: 41 10.1186/s13041-015-0129-7 26170135 PMC4501290

[11] Pasternak SH, Bagshaw RD, Guiral M, Zhang S, Ackerley CA, Pak BJ, et al Presenilin-1, nicastrin, amyloid precursor protein, and gamma-secretase activity are co-localized in the lysosomal membrane. J Biol Chem. 2003;278: 26687–26694. 10.1074/jbc.M212192200 12736250

[12] Bagshaw RD, Pasternak SH, Mahuran DJ, Callahan JW. Nicastrin is a resident lysosomal membrane protein. Biochemical and Biophysical Research Communications. 2003;300: 615–618. 12507492 10.1016/s0006-291x(02)02865-6

[13] Pasternak SH, Callahan JW, Mahuran DJ. The role of the endosomal/lysosomal system in amyloid-beta production and the pathophysiology of Alzheimer's disease: reexamining the spatial paradox from a lysosomal perspective. J Alzheimers Dis. 2004;6: 53–65. 15004328 10.3233/jad-2004-6107

[14] Caporaso GL, Gandy SE, Buxbaum JD, Greengard P. Chloroquine inhibits intracellular degradation but not secretion of Alzheimer beta/A4 amyloid precursor protein. Proc Natl Acad Sci USA. 1992;89: 2252–2256. 1549591 10.1073/pnas.89.6.2252 PMC48635

[15] Haass C, Hung AY, Schlossmacher MG, Teplow DB, Selkoe DJ. beta-Amyloid peptide and a 3-kDa fragment are derived by distinct cellular mechanisms. J Biol Chem. 1993;268: 3021–3024. 8428976

[16] Schrader-Fischer G, Paganetti PA. Effect of alkalizing agents on the processing of the beta-amyloid precursor protein. Brain Research. 1996;716: 91–100. 10.1016/0006-8993(96)00002-9 8738224

[17] Hirschberg K, Miller CM, Ellenberg J, Presley JF, Siggia ED, Phair RD, et al Kinetic analysis of secretory protein traffic and characterization of golgi to plasma membrane transport intermediates in living cells. The Journal of Cell Biology. 1998;143: 1485–1503. 9852146 10.1083/jcb.143.6.1485 PMC2132993

[18] Patterson GH, Patterson GH, Lippincott-Schwartz J. A photoactivatable GFP for selective photolabeling of proteins and cells. Science. American Association for the Advancement of Science; 2002;297: 1873–1877. 10.1126/science.1074952 12228718

[19] Patterson GH, Lippincott-Schwartz J. Selective photolabeling of proteins using photoactivatable GFP. Methods. 2004;32: 445–450. 10.1016/j.ymeth.2003.10.006 15003607

[20] Tam JH, Seah C, Pasternak SH. The Amyloid Precursor Protein is rapidly transported from the Golgi apparatus to the lysosome and where it is processed into beta-amyloid. Mol Brain. BioMed Central Ltd; 2014;7: 54 10.1186/s13041-014-0054-1 25085554 PMC4237969

[21] Bonifacino JS, Traub LM. Signals for sorting of transmembrane proteins to endosomes and lysosomes. Annu Rev Biochem. 2003;72: 395–447. 10.1146/annurev.biochem.72.121801.161800 12651740

[22] Burgos PV, Mardones GA, Rojas AL, daSilva LLP, Prabhu Y, Hurley JH, et al Sorting of the Alzheimer's disease amyloid precursor protein mediated by the AP-4 complex. Developmental Cell. 2010;18: 425–436. 10.1016/j.devcel.2010.01.015 20230749 PMC2841041

[23] Icking A, Amaddii M, Ruonala M, Höning S, Tikkanen R. Polarized Transport of Alzheimer Amyloid Precursor Protein Is Mediated by Adaptor Protein Complex AP1-1B. Traffic. 2006;8: 285–296. 10.1111/j.1600-0854.2006.00526.x 17319802

[24] Schneider A, Rajendran L, Honsho M, Gralle M, Donnert G, Wouters F, et al Flotillin-Dependent Clustering of the Amyloid Precursor Protein Regulates Its Endocytosis and Amyloidogenic Processing in Neurons. Journal of Neuroscience. 2008;28: 2874–2882. 10.1523/JNEUROSCI.5345-07.2008 18337418 PMC6670660

[25] Davis CG, Lehrman MA, Russell DW, Anderson RG, Brown MS, Goldstein JL. The J.D. mutation in familial hypercholesterolemia: amino acid substitution in cytoplasmic domain impedes internalization of LDL receptors. Cell. 1986;45: 15–24. 3955657 10.1016/0092-8674(86)90533-7

[26] Chen WJ, Goldstein JL, Brown MS. NPXY, a sequence often found in cytoplasmic tails, is required for coated pit-mediated internalization of the low density lipoprotein receptor. J Biol Chem. 1990;265: 3116–3123. 1968060

[27] Suzuki T, Nairn AC, Gandy SE, Greengard P. Phosphorylation of Alzheimer amyloid precursor protein by protein kinase C. NSC. 1992;48: 755–761. 10.1016/0306-4522(92)90264-3 1630623

[28] Gandy S, Czernik AJ, Greengard P. Phosphorylation of Alzheimer disease amyloid precursor peptide by protein kinase C and Ca2+/calmodulin-dependent protein kinase II. Proc Natl Acad Sci USA. 1988;85: 6218–6221. 3137567 10.1073/pnas.85.16.6218 PMC281937

[29] Pedersen WA, Kloczewiak MA, Blusztajn JK. Amyloid beta-protein reduces acetylcholine synthesis in a cell line derived from cholinergic neurons of the basal forebrain. Proc Natl Acad Sci USA. 1996;93: 8068–8071. 8755604 10.1073/pnas.93.15.8068 PMC38876

[30] Hammond DN, Wainer BH, Tonsgard JH, Heller A. Neuronal properties of clonal hybrid cell lines derived from central cholinergic neurons. Science. American Association for the Advancement of Science; 1986;234: 1237–1240. 10.1126/science.3775382 3775382

[31] Le WD, Xie WJ, Kong R, Appel SH. Beta-amyloid-induced neurotoxicity of a hybrid septal cell line associated with increased tau phosphorylation and expression of beta-amyloid precursor protein. Journal of Neurochemistry. 1997;69: 978–985. 9282919 10.1046/j.1471-4159.1997.69030978.x

[32] Tam JH, Pasternak SH. Imaging the Intracellular Trafficking of APP with Photoactivatable GFP. J Vis Exp. 2015;: 1–9. 10.3791/53153 26555118 PMC4692657

[33] Lai A, Gibson A, Hopkins CR, Trowbridge IS. Signal-dependent trafficking of beta-amyloid precursor protein-transferrin receptor chimeras in madin-darby canine kidney cells. J Biol Chem. 1998;273: 3732–3739. 9452505 10.1074/jbc.273.6.3732

[34] Cai J, Chen Z, Ruan Q, Han S, Liu L, Qi X, et al -Secretase and Presenilin Mediate Cleavage and Phosphorylation of Vascular Endothelial Growth Factor Receptor-1. Journal of Biological Chemistry. 2011;286: 42514–42523. 10.1074/jbc.M111.296590 22016384 PMC3234916

[35] Greenberg JI, Shields DJ, Barillas SG, Acevedo LM, Murphy E, Huang J, et al A role for VEGF as a negative regulator of pericyte function and vessel maturation. Nature. 2008;456: 809–813. 10.1038/nature07424 18997771 PMC2605188

[36] Gajadhar A, Guha A. A proximity ligation assay using transiently transfected, epitope-tagged proteins: application for in situ detection of dimerized receptor tyrosine kinases. BioTechniques. 2010;48: 145–152. 10.2144/000113354 20359299

[37] Lee M-S, Kao S-C, Lemere CA, Xia W, Tseng H-C, Zhou Y, et al APP processing is regulated by cytoplasmic phosphorylation. Journal of Cell Biology. 2003;163: 83–95. 10.1083/jcb.200301115 14557249 PMC2173445

[38] Vieira SI, Rebelo S, Esselmann H, Wiltfang J, Lah J, Lane R, et al Retrieval of the Alzheimer's amyloid precursor protein from the endosome to the TGN is S655 phosphorylation state-dependent and retromer-mediated. Molecular Neurodegeneration. 2010;5: 40 10.1186/1750-1326-5-40 20937087 PMC2994555

[39] Vieira SI, Rebelo S, Domingues SC, Cruz e Silva EF, Cruz e Silva OAB. S655 phosphorylation enhances APP secretory traffic. Mol Cell Biochem. 2009;328: 145–154. 10.1007/s11010-009-0084-7 19381782

[40] Skovronsky DM, Moore DB, Milla ME, Doms RW, Lee VM. Protein kinase C-dependent alpha-secretase competes with beta-secretase for cleavage of amyloid-beta precursor protein in the trans-golgi network. Journal of Biological Chemistry. 2000;275: 2568–2575. 10.1074/jbc.275.4.2568 10644715

[41] Lammich S, Kojro E, Postina R, Gilbert S, Pfeiffer R, Jasionowski M, et al Constitutive and regulated alpha-secretase cleavage of Alzheimer's amyloid precursor protein by a disintegrin metalloprotease. Proc Natl Acad Sci USA. 1999;96: 3922–3927. 10097139 10.1073/pnas.96.7.3922 PMC22396

[42] Benussi L, Govoni S, Gasparini L, Binetti G, Trabucchi M, Bianchetti A, et al Specific role for protein kinase C alpha in the constitutive and regulated secretion of amyloid precursor protein in human skin fibroblasts. Neuroscience Letters. 1998;240: 97–101. 9486481 10.1016/s0304-3940(97)00894-x

[43] Kinouchi T, Sorimachi H, Maruyama K, Mizuno K, Ohno S, Ishiura S, et al Conventional protein kinase C (PKC)-alpha and novel PKC epsilon, but not -delta, increase the secretion of an N-terminal fragment of Alzheimer's disease amyloid precursor protein from PKC cDNA transfected 3Y1 fibroblasts. FEBS Letters. 1995;364: 203–206. 7750571 10.1016/0014-5793(95)00392-m

[44] Hongpaisan J, Sun M-K, Alkon DL. PKC ε activation prevents synaptic loss, Aβ elevation, and cognitive deficits in Alzheimer's disease transgenic mice. Journal of Neuroscience. 2011;31: 630–643. 10.1523/JNEUROSCI.5209-10.2011 21228172 PMC6623433

[45] Yeon SW, Jung MW, Ha MJ, Kim SU, Huh K, Savage MJ, et al Blockade of PKCϵ Activation Attenuates Phorbol Ester-Induced Increase of α-Secretase-Derived Secreted Form of Amyloid Precursor Protein. Biochemical and Biophysical Research Communications. 2001;280: 782–787. 10.1006/bbrc.2000.4181 11162589

[46] Nelson TJ, Cui C, Luo Y, Alkon DL. Reduction of beta-amyloid levels by novel protein kinase C(epsilon) activators. J Biol Chem. 2009;284: 34514–34521. 10.1074/jbc.M109.016683 19850930 PMC2787312

[47] Kanno T, Yamamoto H, Yaguchi T, Hi R, Mukasa T, Fujikawa H, et al The linoleic acid derivative DCP-LA selectively activates PKC-epsilon, possibly binding to the phosphatidylserine binding site. The Journal of Lipid Research. American Society for Biochemistry and Molecular Biology; 2006;47: 1146–1156. 10.1194/jlr.M500329-JLR200 16520488

[48] Poulsen E, Larsen A, Zollo A, Jørgensen A, Sanggaard K, Enghild J, et al New Insights to Clathrin and Adaptor Protein 2 for the Design and Development of Therapeutic Strategies. IJMS. 2015;16: 29446–29453. 10.3390/ijms161226181 26690411 PMC4691124

[49] Vieira SI, Rebelo S, Esselmann H, Wiltfang J, Lah J, Lane R, et al Retrieval of the Alzheimer's amyloid precursor protein from the endosome to the TGN is S655 phosphorylation state-dependent and retromer-mediated. Molecular Neurodegeneration. 2010;5: 40 10.1186/1750-1326-5-40 20937087 PMC2994555

[50] Bonifacino JS, Hurley JH. Retromer. Current Opinion in Cell Biology. 2008;20: 427–436. 10.1016/j.ceb.2008.03.009 18472259 PMC2833274

[51] Gabuzda D, Busciglio J, Yankner BA. Inhibition of beta-amyloid production by activation of protein kinase C. Journal of Neurochemistry. 1993;61: 2326–2329. 10.1111/j.1471-4159.1993.tb07479.x 8245986

[52] Lanni C, Mazzucchelli M, Porrello E, Govoni S, Racchi M. Differential involvement of protein kinase C alpha and epsilon in the regulated secretion of soluble amyloid precursor protein. European Journal of Biochemistry. 2004;271: 3068–3075. 10.1111/j.1432-1033.2004.04240.x 15233804

[53] Racchi M, Mazzucchelli M, Pascale A, Sironi M, Govoni S. Role of protein kinase C|[alpha]| in the regulated secretion of the amyloid precursor protein. Mol Psychiatry. Nature Publishing Group; 2003;8: 209–216. 10.1038/sj.mp.4001204 12610653

[54] Khan TK, Khan TK, Sen A, Sen A, Hongpaisan J, Lim CS, et al PKCε deficits in Alzheimer's disease brains and skin fibroblasts. J Alzheimers Dis. 2015;43: 491–509. 10.3233/JAD-141221 25125477

[55] Chen Z, Krmar RT, Dada L, Efendiev R, Leibiger IB, Pedemonte CH, et al Phosphorylation of adaptor protein-2 mu2 is essential for Na+,K+-ATPase endocytosis in response to either G protein-coupled receptor or reactive oxygen species. 2006;35: 127–132. 10.1165/rcmb.2006-0044OC 16498080 PMC2658693

[56] Lehel C, Olah Z, Jakab G, Anderson WB. Protein kinase C epsilon is localized to the Golgi via its zinc-finger domain and modulates Golgi function. Proc Natl Acad Sci USA. 1995;92: 1406–1410. 7877991 10.1073/pnas.92.5.1406 PMC42528

[57] Ivaska J, Whelan RDH, Watson R, Parker PJ. PKC epsilon controls the traffic of beta1 integrins in motile cells. The EMBO Journal. 2002;21: 3608–3619. 10.1093/emboj/cdf371 12110574 PMC126116

[58] Ivaska J, Vuoriluoto K, Huovinen T, Izawa I, Inagaki M, Parker PJ. PKCepsilon-mediated phosphorylation of vimentin controls integrin recycling and motility. The EMBO Journal. 2005;24: 3834–3845. 10.1038/sj.emboj.7600847 16270034 PMC1283946

[59] Saraceno C, Marcello E, Di Marino D, Borroni B, Claeysen S, Perroy J, et al SAP97-mediated ADAM10 trafficking from Golgi outposts depends on PKC phosphorylation. Cell Death Dis. 2014;5: e1547 10.1038/cddis.2014.492 25429624 PMC4260750

[60] Ohtsu H, Dempsey PJ, Eguchi S. ADAMs as mediators of EGF receptor transactivation by G protein-coupled receptors. Am J Physiol, Cell Physiol. 2006;291: C1–10. 10.1152/ajpcell.00620.2005 16769815

[61] Lemjabbar-Alaoui H, Sidhu SS, Mengistab A, Gallup M, Basbaum C. TACE/ADAM-17 Phosphorylation by PKC-Epsilon Mediates Premalignant Changes in Tobacco Smoke-Exposed Lung Cells. Rich B, editor. PLoS ONE. 2011;6: e17489 10.1371/journal.pone.0017489.g008 21423656 PMC3057966

[62] Retromer in Alzheimer disease, Parkinson disease and other neurological disorders. 2015;16: 126–132. 10.1038/nrn3896 25669742

