# Supplementary material for: Tyrosine Binding Protein Sites Regulate the Intracellular Trafficking and Processing of Amyloid Precursor Protein through a Novel Lysosome-Directed Pathway
Source: PLoS One. 2016 Oct 24;11(10):e0161445. doi: 10.1371/journal.pone.0161445 (PMC5077117; doi:10.1371/journal.pone.0161445)

Supplemental Figure 1.
PKCε activation does not control the trafficking of APP with S711E or S711A mutations.
Tam et al. 2016

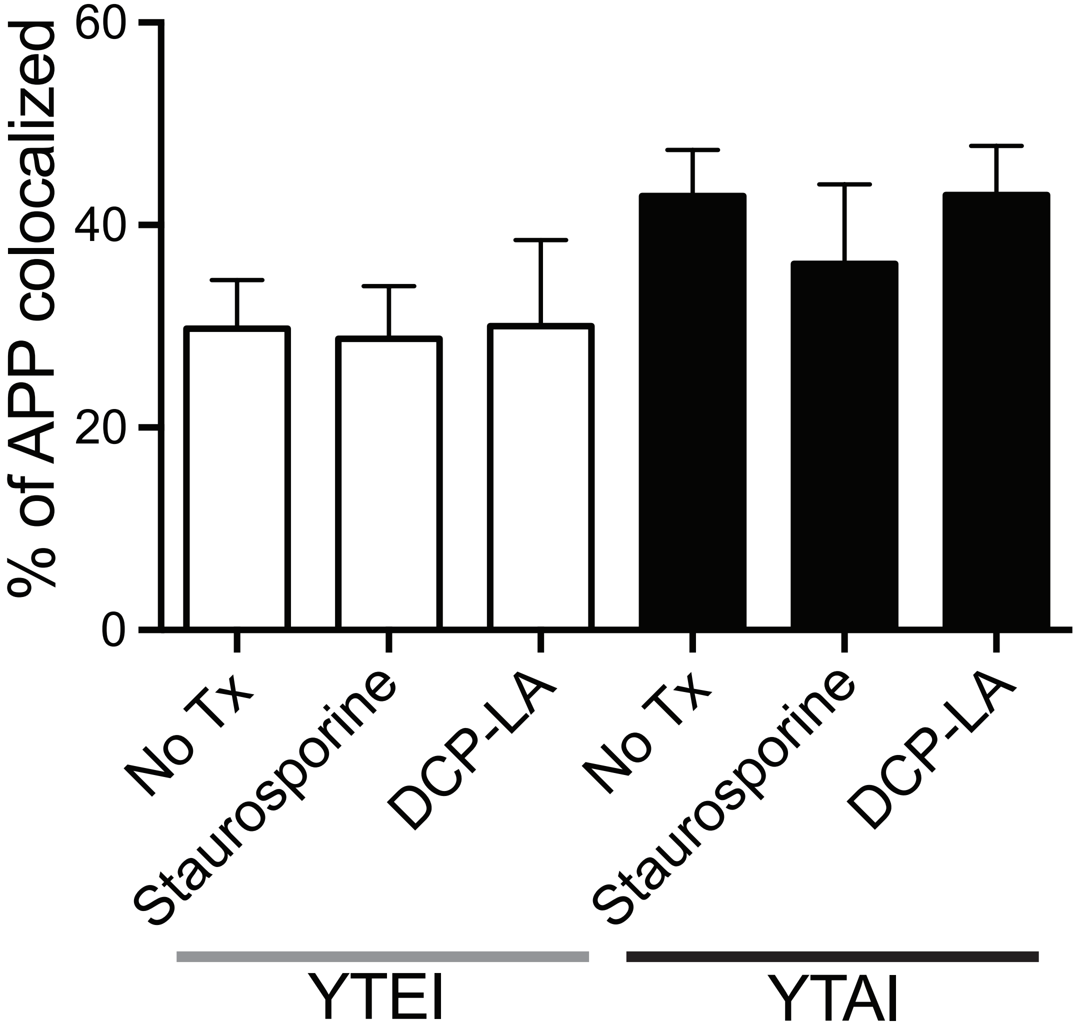

Supplement: S1 Fig — Cells were transfected with plasmids expressing S711E or S711A and a marker for lysosomes (LAMP1-mRFP) Before photoactivation, cells were pretreated with DCP-LA or staurosporine, as described earlier. APP was photo-activated in the Golgi with 405nm light, alternating with imaging for 15 minutes. The percentage of APP colocalized with either LAMP1 was quantified with Imaris. The percentage of APP colocalized was plotted in Graphpad Prism. Error bars represent SEM. (PDF) [file pone.0161445.s001.pdf]
